# Surgical treatment of thoracic vertebral chondrosarcoma with total en bloc spondylectomy: a case report

**DOI:** 10.1093/jscr/rjag979

**Published:** 2026-10-27

**Authors:** Yudha M Sakti, Zikrina A Lanodiyu, Thomas E Prasetyo, Muhammad I Nurfadhilah, Dyah A P Sari, Muchtar Ridha, Wiwid Santiko, Abirafdi A Darutama, Vylomonicca Anggunio, Ilham A Pratama, Andika S Aji, Kadek E Calisto, Zahra N Adamina, Tedjo Rukmoyo

**Affiliations:** Orthopaedics and Traumatology Department, RSUP Dr. Sardjito Hospital, Sleman, D.I.Yogyakarta 55281, Indonesia; Orthopaedics and Traumatology Department, RSUP Dr. Sardjito Hospital, Sleman, D.I.Yogyakarta 55281, Indonesia; Orthopaedics and Traumatology Department, RSUP Dr. Sardjito Hospital, Sleman, D.I.Yogyakarta 55281, Indonesia; Orthopaedics and Traumatology Department, RSUP Dr. Sardjito Hospital, Sleman, D.I.Yogyakarta 55281, Indonesia; Orthopaedics and Traumatology Department, RSUP Dr. Sardjito Hospital, Sleman, D.I.Yogyakarta 55281, Indonesia; Orthopaedics and Traumatology Department, RSUP Dr. Sardjito Hospital, Sleman, D.I.Yogyakarta 55281, Indonesia; Orthopaedics and Traumatology Department, RSUP Dr. Sardjito Hospital, Sleman, D.I.Yogyakarta 55281, Indonesia; Orthopaedics and Traumatology Department, RSUP Dr. Sardjito Hospital, Sleman, D.I.Yogyakarta 55281, Indonesia; Orthopaedics and Traumatology Department, RSUP Dr. Sardjito Hospital, Sleman, D.I.Yogyakarta 55281, Indonesia; Orthopaedics and Traumatology Department, RSUP Dr. Sardjito Hospital, Sleman, D.I.Yogyakarta 55281, Indonesia; Orthopaedics and Traumatology Department, RSUP Dr. Sardjito Hospital, Sleman, D.I.Yogyakarta 55281, Indonesia; Orthopaedics and Traumatology Department, RSUP Dr. Sardjito Hospital, Sleman, D.I.Yogyakarta 55281, Indonesia; Orthopaedics and Traumatology Department, RSUP Dr. Sardjito Hospital, Sleman, D.I.Yogyakarta 55281, Indonesia; Orthopaedics and Traumatology Department, RSUP Dr. Sardjito Hospital, Sleman, D.I.Yogyakarta 55281, Indonesia

**Keywords:** chondrosarcoma, thoracic spine, total en bloc spondylectomy, chemoresistance, radioresistance

## Abstract

Spinal chondrosarcoma is a rare malignant cartilage-forming tumor that is largely resistant to chemotherapy and radiotherapy, making complete surgical resection the cornerstone of treatment. We report a 60-year-old man with recurrent thoracic chondrosarcoma involving the T4–T6 vertebrae following previous intralesional decompression, spinal stabilization, chemotherapy, and radiotherapy. Revision surgery was performed through a posterior approach with implant removal, wide tumor excision, total en bloc spondylectomy (TES), spinal reconstruction using an expandable cage, posterior instrumentation, and spinal fusion. Histopathological examination confirmed recurrent grade II conventional chondrosarcoma. The patient remained neurologically stable after surgery without major perioperative complications. The present case indicates the limitations of intralesional surgery and adjuvant therapy in conventional chondrosarcoma and supports TES as the treatment for complete tumor excision and optimal local control, despite its technical difficulty.

## Introduction

Chondrosarcoma is a malignant primary bone tumor that produces a cartilage matrix. It is most common in adults and occurs in the pelvis or long bones; men are affected two to four times more often than women. Spinal involvement is rare and accounts for only 2%–12% of all chondrosarcomas with the thoracic spine being the most common site followed by the cervical and lumbar spine [1]. Spinal chondrosarcoma presents unique clinical challenges because of its proximity to the spinal cord, major vascular structures, and thoracic viscera. Patients usually present with progressive back pain and neurological deficits may develop secondary to the compression of the spinal canal as the tumor enlarges [2].

Conventional chondrosarcoma is largely resistant to chemotherapy and radiotherapy, which makes complete surgical resection the cornerstone of treatment, unlike many other malignancies [1]. En bloc resection with negative margins is the preferred surgical strategy whenever technically feasible, as it provides better local tumor control, lower recurrence rates, and improved long-term survival compared with intralesional or piecemeal excision [3–5]. However, total en bloc spondylectomy (TES) is technically challenging, requiring careful planning and execution to achieve oncological clearance while minimizing the risk of neurological injury, vascular complications, and postoperative spinal instability.

Evidence guiding surgical management is limited, being largely derived from case reports and small case series, given the rarity of thoracic spinal chondrosarcoma, especially cases involving multiple vertebral levels. We describe an unusual case of chondrosarcoma of the thoracic vertebrae involving T4–T6, initially diagnosed as grade I and recurring as grade II, that was treated successfully with TES and spinal reconstruction. The surgical approach, technical details, and multidisciplinary management for complete resection of the tumor with preservation of neurological function and spinal stability are emphasized.

## Case report

A 60-year-old male patient with a history of working as a truck driver and laborer, presented with progressive numbness of both lower extremities. Symptoms began 2.5 years earlier as sudden-onset numbness in the left leg, gradually progressing to involve the right leg. Initial conservative treatment at a regional hospital was unsuccessful. Two years before the current admission, he underwent posterior decompression consisting of laminectomy, debridement, spinal stabilization, open biopsy, and tissue culture at our institution. Following surgery, he received six cycles of doxorubicin and cisplatin chemotherapy and 33 fractions of adjuvant radiotherapy because of residual tumor. Follow-up magnetic resonance imaging (MRI) demonstrated local recurrence, and the patient was referred back to the Orthopaedic and Traumatology Department for definitive surgical management.

On admission, the patient was fully conscious, ambulatory, and hemodynamically stable. Physical examination revealed no spinal deformity or tenderness. Motor strength was preserved (grade 5/5) in all extremities, whereas sensory examination demonstrated hypoesthesia below the T5 dermatome. Bowel and bladder function were intact, and neurological status was classified as American Spinal Injury Association Impairment Scale grade D.

Laboratory investigations were within normal limits. Plain radiographs demonstrated previous posterior instrumentation with a 36.5° thoracic scoliosis. Thoracic MRI revealed recurrent tumor involving the T4–T6 vertebrae. The diagnosis of recurrent thoracic chondrosarcoma was made based on clinical, radiological, and previous histopathological findings (Fig. 1). A multidisciplinary team planned revision surgery consisting of implant removal, wide tumor excision, TES, and instrumented spinal reconstruction.

**Figure 1:**
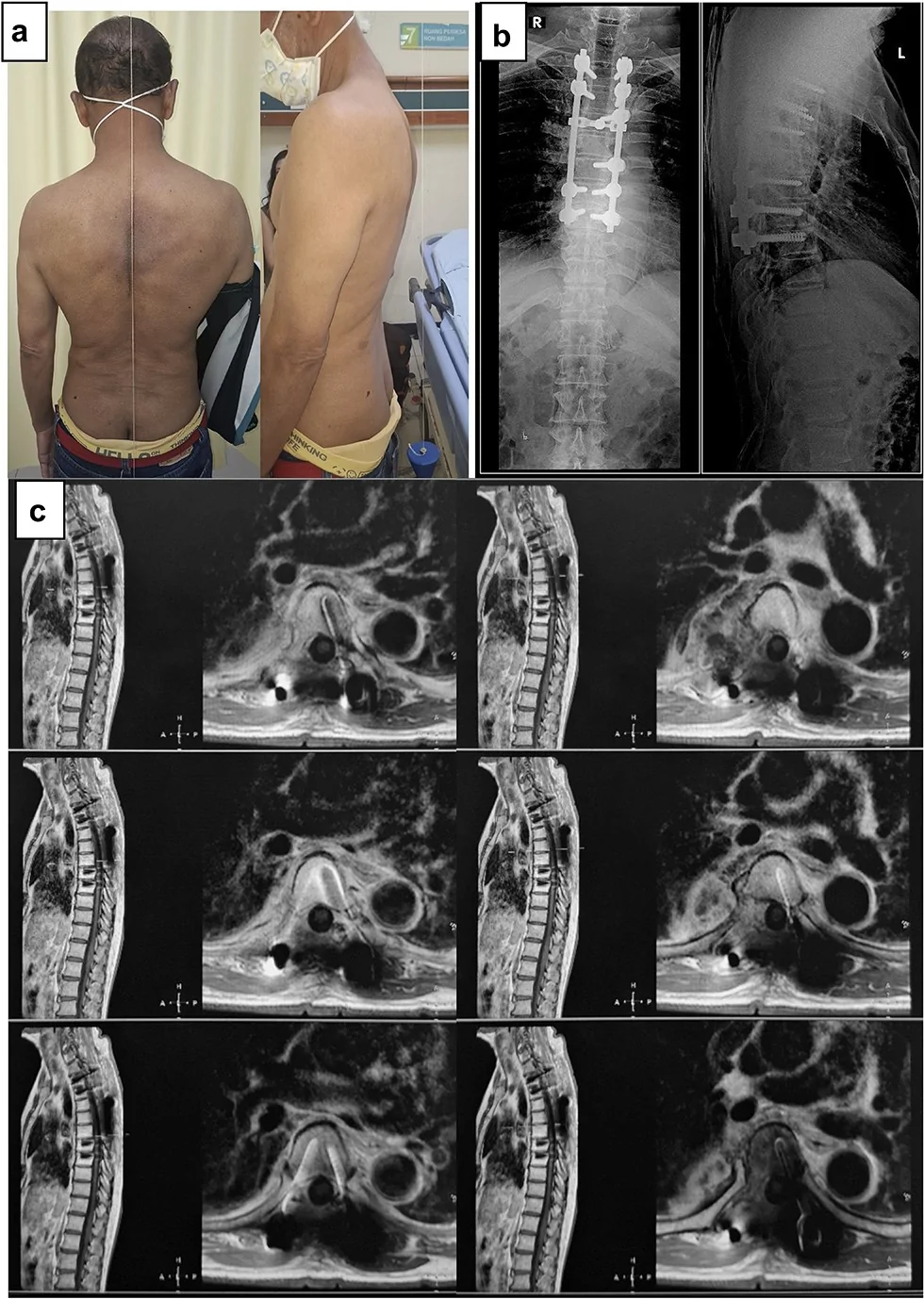
Pre-operative documentation: (a) clinical pictures, (b) thoracic spine X-ray, (c) thoracic spine MRI sequences demonstrating a large, recurrent extramedullary mass spanning the T4–T6 vertebral levels.

Through a posterior midline approach, the previous implants were removed and the tumor margins were identified. A well-circumscribed solid mass involving T4–T6 was excised en bloc, including partial spondylectomy of T3 and total spondylectomy of T4, followed by anterior cage reconstruction and posterior instrumentation with cross-links (Figs 2 and 3).

**Figure 2:**
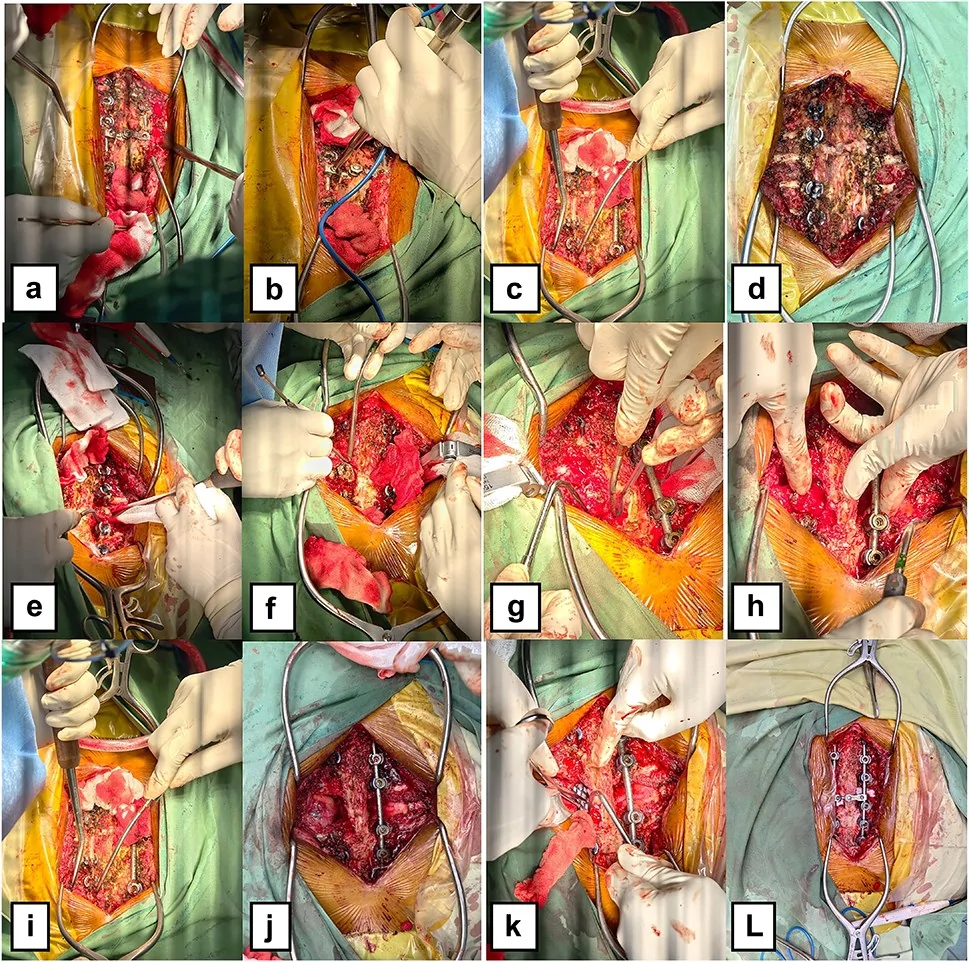
Surgery procedure of removal of the existing posterior implants, wide tumor excision, open biopsy, total spondylectomy, and spinal fusion: (a) posterior median approach, previous implants are visible, (b) removal of crosslink, (c) removal of the first rod for a better approach to the tumor, (d) removal of the second rod, (e and f) en bloc resection of the tumor, (g) reinsertion of the contralateral rod and discectomy of upper and lower discs with curettage, (h) post-resection of the tumor, before removal, (i) rib resection, (j) post tumor excision, (k) insertion of the cage, (l) post fixation and removal of the tumor.

**Figure 3:**
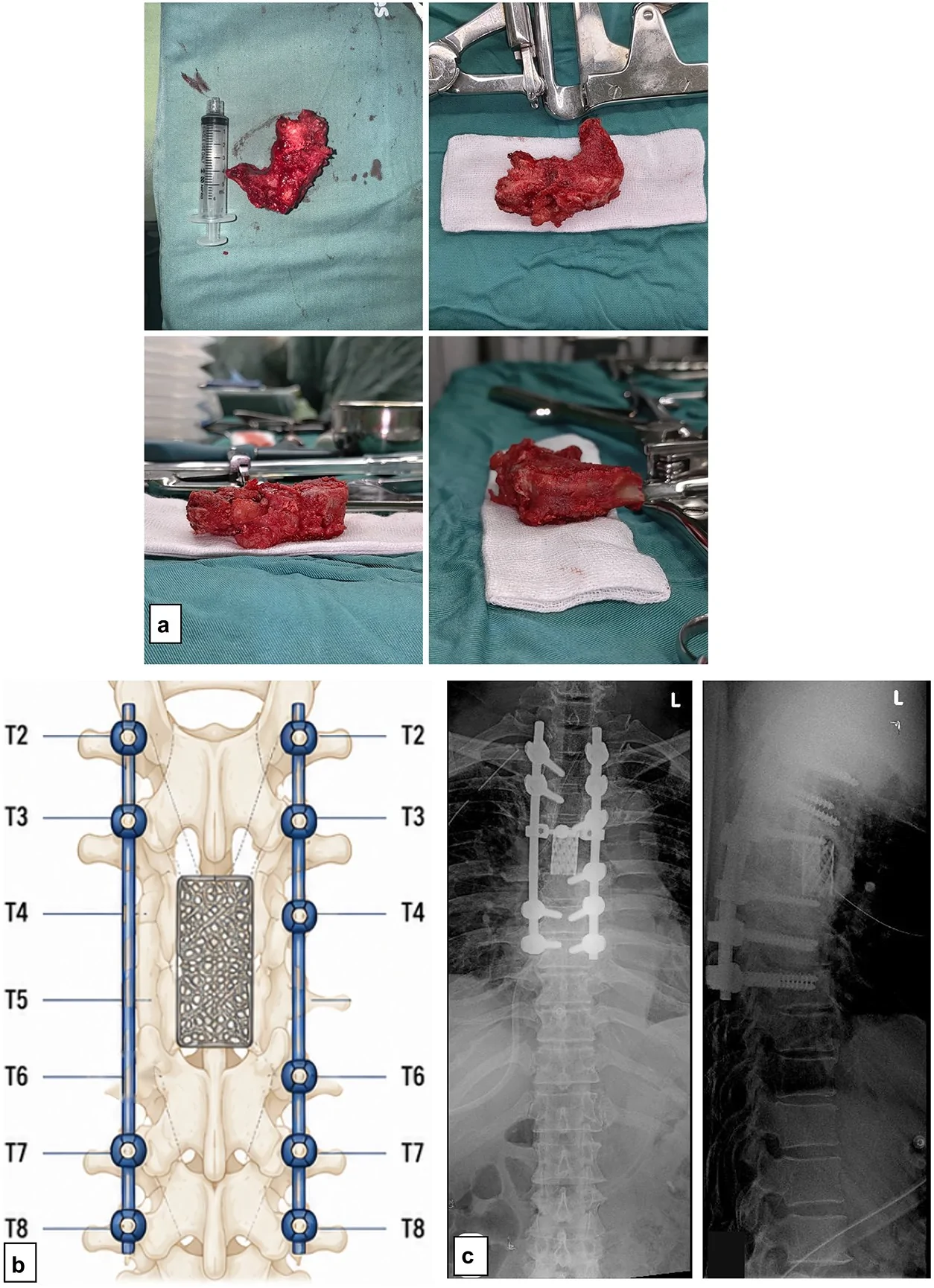
Postoperative documentation: (a) TES of thoracic spine chondrosarcoma, (b) design of cage placement following posterior stabilization after spondylectomy, (c) thoracolumbar X-ray imaging, the T4 thoracic vertebrae body is not visualized due to corpectomy.

Histopathological examination demonstrated a highly cellular conventional chondroid neoplasm with lobulated architecture infiltrating adjacent bone and soft tissue (Fig. 4). Tumor cells showed moderate to marked nuclear pleomorphism, occasional binucleated chondrocytes within lacunae, abundant basophilic chondroid matrix with focal myxoid change and identifiable mitotic figures. These findings confirmed recurrent grade II conventional chondrosarcoma.

**Figure 4:**
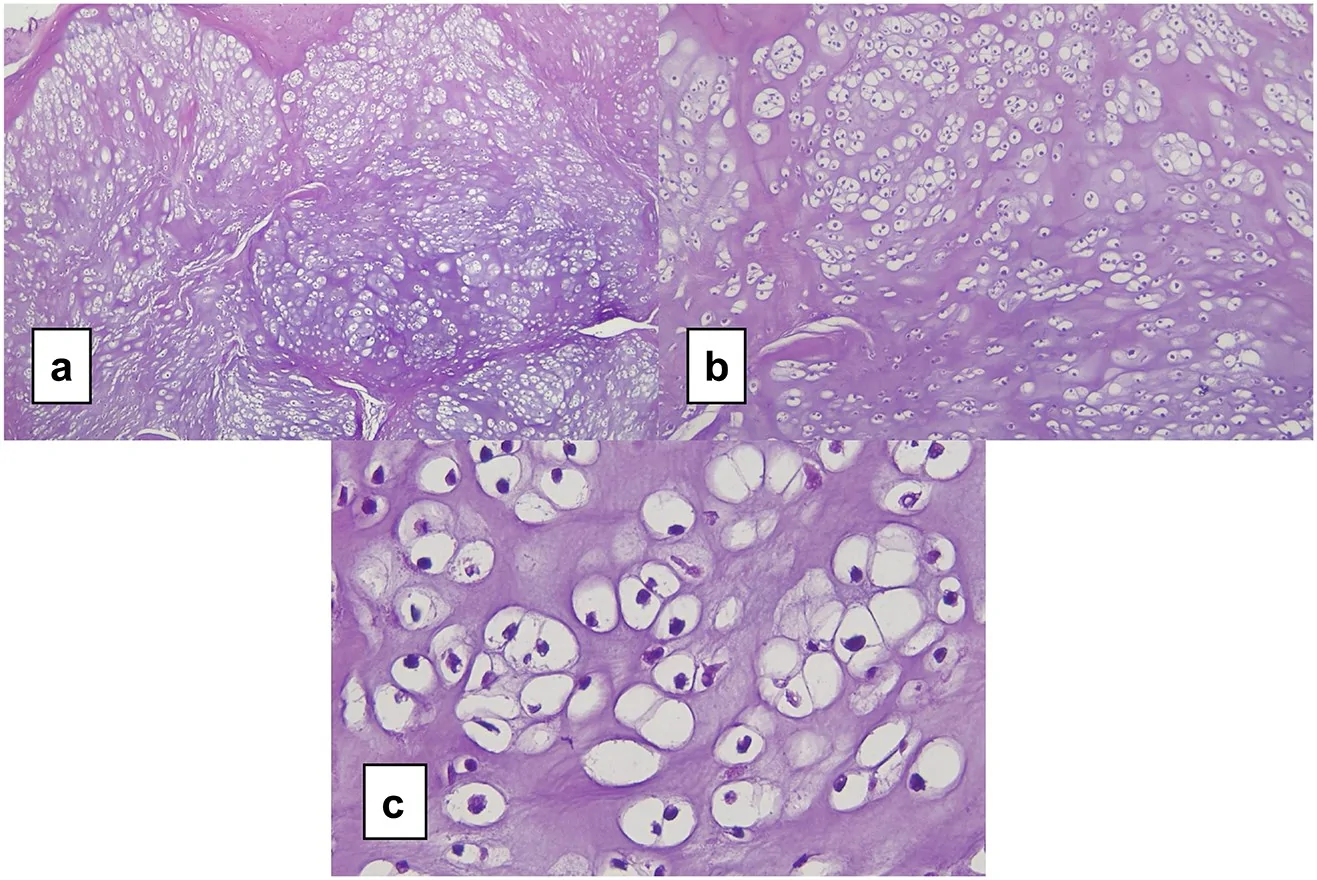
Histopathological finding on (a) 40× magnification, (b) 100× magnification, and (c) 400× magnification. Based on this examination, we found a highly cellular chondroid tissue arranged in lobules, infiltrating adjacent bone, and connective tissue. The tumor cells are pleomorphic, ranging from medium to large in size, with round, oval, and spindle-shaped morphology; some cells are binucleated and located within lacunae. The background consists of a basophilic chondroid matrix with focal myxoid degeneration. Mitotic figures are identified.

The postoperative course was uneventful without major neurological or wound complications. The neurological examination on follow-up demonstrated intact motor strength (5/5) in all extremities with continued hypoesthesia below the T5 dermatome. Deep tendon reflexes were normal, pathological reflexes were absent, and bowel and bladder function remained intact. At 3 months postoperatively, contrast-enhanced computed tomography (CT) of the thoracic spine demonstrated satisfactory postoperative spinal reconstruction with no evidence of residual or recurrent tumor, as shown in (Fig. 5). The patient remained neurologically stable, with preserved motor function and no new neurological deficits.

**Figure 5:**
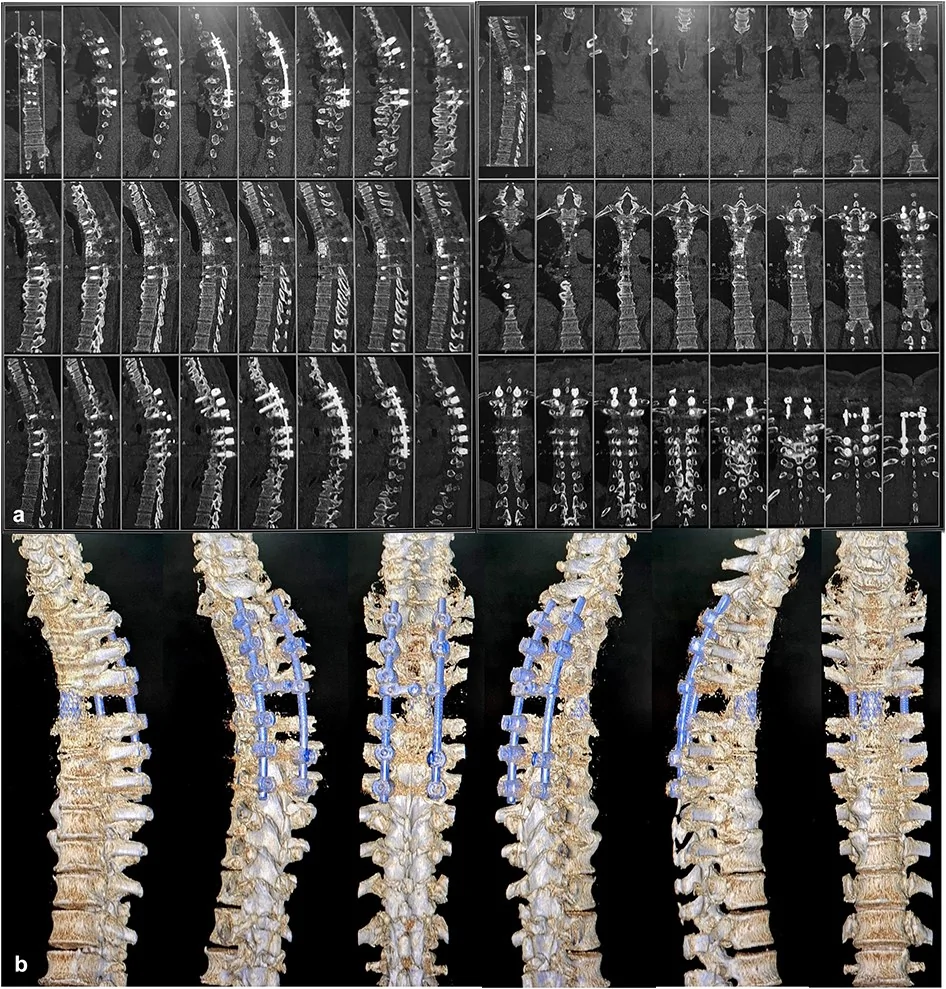
Three-month postoperative contrast-enhanced CT scan of the thoracic spine: (a) multiplanar CT images demonstrating the postoperative spinal reconstruction with posterior instrumentation and cage placement following TES; (b) three-dimensional CT reconstruction demonstrating the posterior instrumentation and satisfactory spinal alignment. No radiological evidence of residual or recurrent tumor was observed.

## Discussion

Primary spinal chondrosarcoma is a rare malignant cartilage-producing tumor that is difficult to treat because of its locally aggressive behavior and poor response to chemotherapy and radiotherapy. This case illustrates recurrence after prior intralesional decompression with adjuvant chemoradiotherapy, with incomplete excision resulting in a high risk of local recurrence. Thus, complete surgical resection is the mainstay of treatment for durable local control.

The prognosis of spinal chondrosarcoma is mainly dictated by histological grade and the adequacy of surgical margins. Higher grade tumors, axial skeletal location, and positive surgical margins are correlated with increased local recurrence and poorer survival [6–8]. Although our patient was initially diagnosed with grade I disease, recurrent histopathology showed progression to grade II conventional chondrosarcoma, underlining the importance of complete oncological resection and long-term surveillance.

Conventional chondrosarcoma is characteristically resistant to chemotherapy and radiotherapy because of its dense extracellular cartilaginous matrix, poor vascularity, hypoxic microenvironment, slow cellular proliferation, and enhanced multidrug resistance mechanisms [6, 9, 10]. Consequently, TES with negative margins is considered the preferred treatment whenever technically feasible, as it provides superior local control and lower recurrence rates compared with intralesional or piecemeal excision [3, 11–13]. However, TES remains one of the most technically demanding spinal oncology procedures because of the need to preserve neural and vascular structures while achieving adequate oncological margins. Previous studies have reported the procedure to be associated with long operative time, significant blood loss and high rates of perioperative complications [8]. In this case, TES allowed complete resection of the tumor and reconstruction of the spine despite prior surgery and adjuvant treatment. The patient was neurologically stable in the post-operative period and histopathology confirmed recurrent grade II conventional chondrosarcoma. This case further adds to the current evidence that TES is the treatment of choice for resectable thoracic spinal chondrosarcoma and emphasizes the importance of multidisciplinary planning to optimize oncological resection, preservation of neurological function and spinal stability.

## Data Availability

This article has no associated data generated and/or analyzed.
